# Association of anti-peptidyl arginine deiminase antibodies with radiographic severity of rheumatoid arthritis in African Americans

**DOI:** 10.1186/s13075-016-1126-7

**Published:** 2016-10-22

**Authors:** Iris Navarro-Millán, Erika Darrah, Andrew O. Westfall, Ted R. Mikuls, Richard J. Reynolds, Maria I. Danila, Jeffrey R. Curtis, CLEAR Investigators, Antony Rosen, S. Louis Bridges

**Affiliations:** 1 University of Alabama at Birmingham, 510 20th Street South, Faculty Office Tower 850, Birmingham, AL 35294-3408 USA; 2 Division of Rheumatology, The Johns Hopkins University, Baltimore, MD USA; 3 VA Nebraska-Western Iowa Health Care System and University of Nebraska Medical Center, Omaha, NE USA

**Keywords:** Anti-PAD4, Rheumatoid arthritis, Radiographic severity, African American

## Abstract

**Background:**

Evidence suggests that the presence of peptidyl arginine deiminase type 4 (PAD4) antibodies is associated with radiographic-severity rheumatoid arthritis (RA) among Caucasian patients. The presence of anti-PAD4 antibodies that were cross-reactivity against PAD3 was associated with more aggressive erosive disease (compared with the presence of anti-PAD4 antibodies without anti-PAD3 crossreactivity) in Caucasian RA patients. The objectives of this study were to determine the prevalence of serum anti-PAD4 and anti-PAD4/PAD3 cross-reactive autoantibodies in African Americans with RA and whether these antibodies associate with radiographic severity and radiographic progression.

**Methods:**

Serum anti-PAD4 and anti-PAD4/PAD3 antibodies were measured by immunoprecipitation, and the temporal trends in titers were analyzed. We compared total radiographic scores among anti-PAD4-positive, anti-PAD4/PAD3-positive, and anti-PAD4-negative patients and used a zero-inflated negative binomial model to determine associations between radiographic severity and antibody status. Logistic regression was used to analyze radiographic progression.

**Results:**

Of 192 African-American patients with RA, 73 % were anti-citrullinated peptide/protein antibody (ACPA)-positive, 46 out of 192 (24 %) of whom had serum anti-PAD4 antibodies. Median (interquartile range) total Sharp van der Heijde radiographic scores were 2 (1–97.5) in ACPA-positive patients and 0 (0–3) in ACPA-negative patients (*P* < 0.001). Of the 46 anti-PAD4-positive patients, 20 had anti-PAD4 antibodies that cross-reacted with PAD3. In patients with early RA, anti-PAD4 and anti-PAD4/PAD3 antibody titers increased over time (*P* = 0.006, *P* = 0.001, respectively). Median (interquartile range) total radiographic scores were higher for anti-PAD4-positive than for anti-PAD4-negative patients (3 (1–115) versus 2 (0–11), respectively; *P* = 0.005). Median (interquartile range) total radiographic score for anti-PAD4/PAD3-positive patients was 76 (3–117) (*P* < 0.001) versus anti-PAD4-negative patients. Only anti-PAD4/PAD3 antibodies associated with radiographic severity (incidence rate ratio = 2.81; 95 % confidence interval 1.23, 6.43).

**Conclusion:**

This analysis suggests that autoantibodies against PAD4 and PAD3 proteins may serve as biomarkers for identifying African-American patients with RA and higher radiographic severity.

**Electronic supplementary material:**

The online version of this article (doi:10.1186/s13075-016-1126-7) contains supplementary material, which is available to authorized users.

## Background

Rheumatoid arthritis (RA) is an autoimmune inflammatory arthritis characterized by the presence of serum autoantibodies, including rheumatoid factor and anti-citrullinated peptide/protein antibodies (ACPAs) [1]. Antibodies to citrulline-containing epitopes in a variety of proteins, including enolase and filaggrin among others, appear to be common (50–60 %) in RA and are relatively specific (>95 %) for the disease [2, 3]. Citrullination, catalyzed by the peptidyl arginine deiminase enzymes (PADs), is a calcium-dependent posttranslational process in which arginine residues are deiminated [4]. In RA, type 4 PAD (PAD4, encoded by *PADI4* on chromosome 1p) is preferentially expressed in hematopoietic cells, contributes to the generation of ACPA-specific substrates, and is also a target of disease-specific autoantibodies [4].

PAD4 has an essential role in RA disease severity and predisposition, with population studies demonstrating genetic associations of *PADI4* with RA, particularly among Asian populations [5]. Among the European ancestry population, autoantibodies to the PAD4 protein were reported to be 42 % sensitive and 99 % specific for RA in a cross-sectional study, and to associate with radiographic severity [6, 7]. A proportion of these anti-PAD4 antibodies have been demonstrated to cross-react with the related protein PAD3, in blocking studies [6]. Anti-PAD4/anti-PAD3 cross-reactivity has been associated with severity of erosive joint disease and interstitial lung disease in RA patients of predominantly European ancestry [6, 8]. Anti-PAD4/PAD3 cross-reactive antibodies are important in the pathogenesis of RA in that they markedly increase the catalytic efficiency of PAD4 by decreasing its requirement for calcium to be active. The presence of serum anti-PAD4/PAD3 antibodies might thus help to identify RA patients who may benefit from aggressive therapy.

The titers of anti-PAD4 autoantibodies were previously analyzed cross-sectionally in non-African populations [6, 8, 9], and studies have determined the prevalence of anti-PAD4 antibodies in established RA to be between 35 and 45 % [7, 10–12]. The prevalence of these antibodies in the prediagnosis and postdiagnosis time periods has been reported to be 18 % and 26 %, respectively, which suggests that the development of these antibodies occurs mainly over time [10]. Information about how these titers change over time may also facilitate determination of the pathophysiologic effect these antibodies have on PAD4 that can subsequently lead to aggressive disease. One study previously revealed that the anti-PAD4 antibodies remain stable over time after treatment with anti-tumor necrosis factor agents [13]. However, the temporal trend in titers of anti-PAD4/PAD3 cross-reactive antibodies has not been analyzed, but is important because of the higher radiographic severity described with these cross-reactive antibodies [8].

Importantly, these autoantibodies have been studied in detail in RA patients of European ancestry and in Native Americans with RA [8, 9], but not in patients of African-American ethnicity. It remains unknown whether these antibodies are highly prevalent among African Americans, and whether their presence is associated with erosive disease in the same way it is among patients of other ethnicities [6, 9]. The strong association of the HLA-DRB1 locus with ACPA-positive RA provides a rationale for examining the anti-PAD4 response in African Americans with RA [14]. Notably, we have recently shown subtle differences in the association of HLA-DRB1 alleles with RA in African Americans [15]. Furthermore, not all non-MHC region risk loci identified in European or Asian RA populations are shared with RA in persons of African ancestry, further justifying the need for research on African Americans with RA [16]. The objectives of this study are therefore: to determine the prevalence of serum anti-PAD4 antibodies (including anti-PAD4/PAD3 cross-reactive antibodies) in African Americans with RA; to describe the temporal changes in anti-PAD4 and anti-PAD4/PAD3 cross-reactive antibody titers; and to compare the radiographic score and radiographic progression of anti-PAD4-positive and anti-PAD4/PAD3-positive African-American patients with those of patients lacking these antibodies.

## Methods

### Patients

**Fig. 1:**
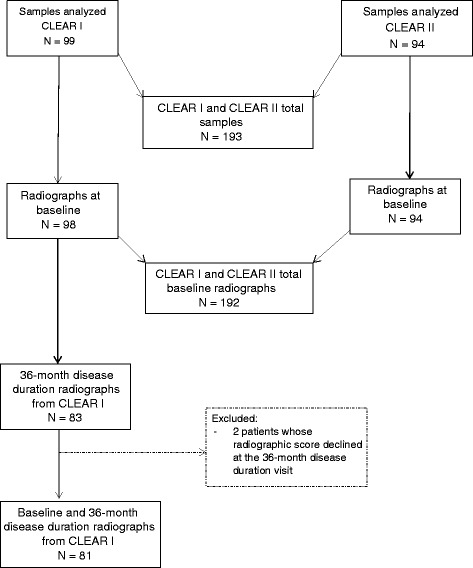
Blood samples and radiographs of patients with RA from the CLEAR I and CLEAR II cohorts. CLEAR I is a longitudinal registry of African-American patients with early RA. CLEAR II is a cross-sectional registry of African-American patients with established RA. *CLEAR* Consortium for the Longitudinal Evaluation of African Americans with Early Rheumatoid Arthritis

In all, 192 participants in the Consortium for the Longitudinal Evaluation of African Americans with Early Rheumatoid Arthritis (CLEAR) Registry were included in this study (Fig. 1). This group of patients has been the subject of multiple reports, with radiographic findings described previously [17]. The CLEAR Registry enrolled patients with RA from 2000 to 2006 (CLEAR I) and from 2006 to 2012 (CLEAR II) as defined by the revised 1987 American College of Rheumatology (ACR) criteria [18], age ≥ 19 years, and self-defining as African American. CLEAR I was a longitudinal registry enrolling African Americans with early RA (disease duration < 2 years at the time of enrollment) with comprehensive demographic, clinical, and radiographic data at baseline (time of enrollment in the cohort) and approximately 36 months from disease onset. CLEAR II enrolled African Americans with RA (without limits of disease duration) at a one-time visit (cross-sectional approach). Subjects enrolled in CLEAR I were not eligible for enrollment in CLEAR II, so there is no overlap between the patients in CLEAR I and CLEAR II.

### Variables

Demographic data, smoking status, medication use, and the number of swollen and tender joints were collected and assessed during the enrollment and follow-up visits. ACPA (IgG) titers were measured in baseline serum, as reported previously [19], using a commercially available second-generation (anti-cyclic citrullinated peptide 2 (anti-CCP2)) enzyme-linked immunosorbent assay (ELISA) kit (Diastat; Axis-Shield Diagnostics, Dundee, UK) using a cutoff value for positivity of ≥5 IU/ml.

CLEAR I patients had radiographs of hands/wrists (posteroanterior views) and feet (anteroposterior views) at baseline (<2 years of disease duration) and approximately 36 months from disease onset. CLEAR II patients had radiographs at the time of the single-study visit. Radiographic scores were assessed using the modified Sharp/van der Heidje score (total radiographic score = total bone erosions and joint space narrowing) as described previously [20].

CLEAR I was an early RA cohort established in 2000. At that time, the use of biologic agents was in the early stages and the majority of the patients in this cohort were on methotrexate without biologic agents. Examination of the contribution of several variables such as age, gender, or medication use revealed these were not significantly associated with radiographic severity or progression in bivariate analysis; hence these variables were not included in the multivariable model. C-reactive protein (CRP) was not included in the multivariable model because measurements were available only at a limited number of time points and thus did not substantively aid the assessment of disease activity over the study period.

### Exposure and comparator

The main exposure of interest was the presence of anti-PAD4 antibodies and anti-PAD4/PAD3 cross-reactive antibodies. The main comparator was anti-PAD4-negative patients.

### Autoantibody-detection assays

The presence of anti-PAD4 only antibodies and anti-PAD4/PAD3 cross-reactive antibodies was determined by immunoprecipitation as described previously [6]. To detect these antibodies we employed a two-tier strategy to first identify all patients in the cohort who had circulating anti-PAD4 antibodies, and then test the anti-PAD4-positive patients for cross-reactivity to PAD3. Briefly, 1 μl of serum was incubated with in-vitro transcribed and translated S^35^-labeled PAD4 protein for 1 hour at 4 °C in NP-40 lysis buffer/0.2 % bovine serum albumin. Protein A agarose beads were then added to immunoprecipitate antigen–antibody complexes. After washes with NP-40 lysis buffer, samples were boiled in SDS gel buffer and resolved by SDS-PAGE. Immunoprecipitated S^35^-labeled PAD4 was visualized by radiography and quantified by densitometry. The densitometry values were normalized to a serum with known high-titer anti-PAD4 autoantibodies, and serum values with normalized anti-PAD4 IU ≥ 0.02 were considered positive. Anti-PAD4 antibody-positive patients were further screened for antibody cross-reactivity to S^35^-labeled PAD3. Anti-PAD3 antibody immunoprecipitation was performed as already described, and samples with normalized anti-PAD3 antibody IU ≥ 0.01 were considered positive. Serum samples with anti-PAD3 or anti-PAD4 antibody IU > 0.2 were considered medium to high titer, and those samples with IU < 0.2 were considered low titer.

### Outcome measures

The main outcome measures were total radiographic score (total score at baseline analyzed cross-sectionally for CLEAR I and CLEAR II combined) and radiographic progression (analyzed only for CLEAR I). For the cross-sectional analysis, radiographs were categorized as having any damage (total radiographic score > 0) or no damage (total radiographic score = 0). Overall progression of radiographic scores in CLEAR I was defined by any unit increase in the total radiographic score using the modified Sharp/van der Heijde score divided by the time elapsed between the enrollment radiographs to those obtained at the 36-month disease duration visit.

A total of 81 CLEAR I patients were analyzed for radiographic progression. This outcome was dichotomized as no radiographic progression or any radiographic progression (total radiographic score of 0 or >0, respectively).

### Statistical analysis

We calculated the prevalence of anti-PAD4 and anti-PAD4/PAD3 antibodies at the time of enrollment in CLEAR. We described the trends (changes from a positive titer to a negative titer status, and vice versa) of anti-PAD4 and anti-PAD4/anti-PAD3 cross-reactive antibody titers from baseline to 36-month disease duration visit using the CLEAR I registry. We also described the relationship and correlation of these titers with RA disease duration. The nonparametric Wilcoxon signed rank test was used to determine whether the changes in these titers were different from 0.

We determined the proportion of patients with radiographic damage (categorical measure) among both cohorts combined (*N* = 192) and among those who were anti-PAD4-positive, anti-PAD4/PAD3-positive, or anti-PAD4-negative. We compared radiographic scores in anti-PAD4-positive and anti-PAD4-negative patients; in anti-PAD4/PAD3-positive and anti-PAD4-negative patients; and in anti-PAD4/PAD3-positive and anti-PAD4-positive patients. Categorical measures were compared using Fisher’s exact tests. Total radiographic scores (continuous measure) were analyzed by the nonparametric Wilcoxon rank sum test, where appropriate.

Because radiographic scores are a count, and given the large number of total radiographic scores of 0, we used zero-inflated negative binomial models to determine the association between anti-PAD4 positivity or anti-PAD4/PAD3 positivity with higher radiographic scores (cross-sectional analysis). Results of these models are reported as the incidence rate ratio (IRR) [21]. 100 % of patients who were anti-PAD3-positive were also anti-PAD4-positive; and 91 % of patients who were anti-PAD4-positive were also ACPA-positive. Thus, to avoid issues with collinearity, the effects of ACPA, anti-PAD4, and anti-PAD4/PAD3 antibody positivity were each evaluated in separate models.

To evaluate radiographic progression, we used total radiographic scores from baseline and from the subsequent visit in CLEAR 1 only. We performed descriptive statistics for CLEAR 1 data prior to building the multivariable logistic regression models used to describe the association between ACPA, anti-PAD4, or anti-PAD4/PAD3 positivity with radiographic progression. The outcome was modeled as change >0 versus change of 0 (no change) in total radiographic score.

## Results

There were 192 patients, 146 were anti-PAD4 antibody-negative and 46 were anti-PAD4 antibody-positive. The mean age of patients was 55 years, mean disease duration was 8 years (for CLEAR I and CLEAR II combined), 86 % of patients were female, and 73 % of patients were ACPA-positive (Table 1). Figure 1 shows the flow diagram for the population of this study. The prevalence of anti-PAD4 antibodies at the time of enrollment in CLEAR was 24 % (*N* = 46), and 91 % of patients who were anti-PAD4-positive were also ACPA-positive. The prevalence of anti-PAD4/PAD3 was 10 % (*N* = 20). Of the anti-PAD4-positive patients (*N* = 46), 83 % had radiographic damage (total radiographic score > 0) at baseline, compared with 63 % in the anti-PAD4-negative group (*N* = 146; *P* = 0.02). Median (interquartile range (IQR)) radiographic scores for the anti-PAD4-positive (*N* = 46) and anti-PAD4-negative (*N* = 146) patients were 3 (1–115) versus 2 (0–11), respectively (*P* = 0.005).

**Table 1:** CLEAR I and CLEAR II RA patient characteristics at the time of enrollment in the registries and stratification by anti-PAD4 and anti-PAD4/PAD3 positivity

	All patients	Anti-PAD4-negative (P0)	Anti-PAD4 only (P4)	Anti-PAD4/PAD3 (XR)	*P* value		
Demographic and RA-related variable	(*N* = 192)	(*N* = 146)	(*N* = 26)	(*N* = 20)	P4 vs P0	XR vs P0	XR vs P4
Age (years), mean (SD)	55 (12.2)	54 (12.1)	55 (13.9)	59 (8.6)	0.755	0.077	0.288
Female, %	86	85	96	80	0.207	0.523	0.151
Disease duration (years), mean (SD)	8.1 (10.8)	7.2 (10.5)	10.4 (11.2)	12.8 (11.6)	**0.029**	**0.004**	0.386
+anti-CCP, %	73	67	88	95	**0.035**	**0.008**	0.622
Total radiographic score,^a^ median (IQR)	2 (0–57)	2 (0–11)	2 (1–104)	76 (3–117)	0.231	**0.001**	0.082
CRP (mg/dl)	14 (36)	16 (40)	8 (10)	9 (13)	1.000	0.391	0.598
Smoking, %					0.387	0.183	0.852
Current	56	33	19	15			
Former	45	21	27	35			
Never	92	46	54	50			
Medications, %							
Methotrexate	63	63	69	85	0.660	0.080	0.302
HCQ	29	29	34	20	0.645	0.441	0.336
Sulfasalazine	8	8	8	5	1.000	1.000	1.000
Leflunomide	5	5	0	5	0.600	1.000	0.435
Infliximab	4	4	0	10	0.593	0.248	0.184
Etanercept	4	4	4	10	1.000	0.248	0.572

Categorical variables were tested using Fisher’s exact tests, and continuous variables were tested using Wilcoxon rank sum tests

^a^Total joint erosion and joint space narrowing

*CLEAR* Consortium for the Longitudinal Evaluation of African Americans with Early Rheumatoid Arthritis, *RA* rheumatoid arthritis, *PAD* peptidyl arginine deiminase enzyme, *CRP* C-reactive protein, *HCQ* hydroxychloroquine, *IQR* interquartile range

*P* values less than 0.05 are shown in bold

RA disease duration was longer among patients who were positive for anti-PAD4 antibodies only (*N* = 26) and those with cross-reactive anti-PAD4/PAD3 antibodies (*N* = 20) than in patients who were negative for either of these antibodies (*N* = 146) (Table 1). The median (IQR) radiographic score for the anti-PAD4/PAD3-positive patients (*N* = 20) was 76 (3–117), which was significantly different from the total radiographic score of the anti-PAD4-negative patients (*N* = 146) (*P* < 0.001) (Table 1). The median (IQR) total radiographic score for patients only positive for anti-PAD4 antibodies (*N* = 26) was 2 (1–104), which was not statistically different from those with anti-PAD4/PAD3 cross-reactive antibodies (*N* = 20) (*P* = 0.073) (Table 1).

**Fig. 2:**
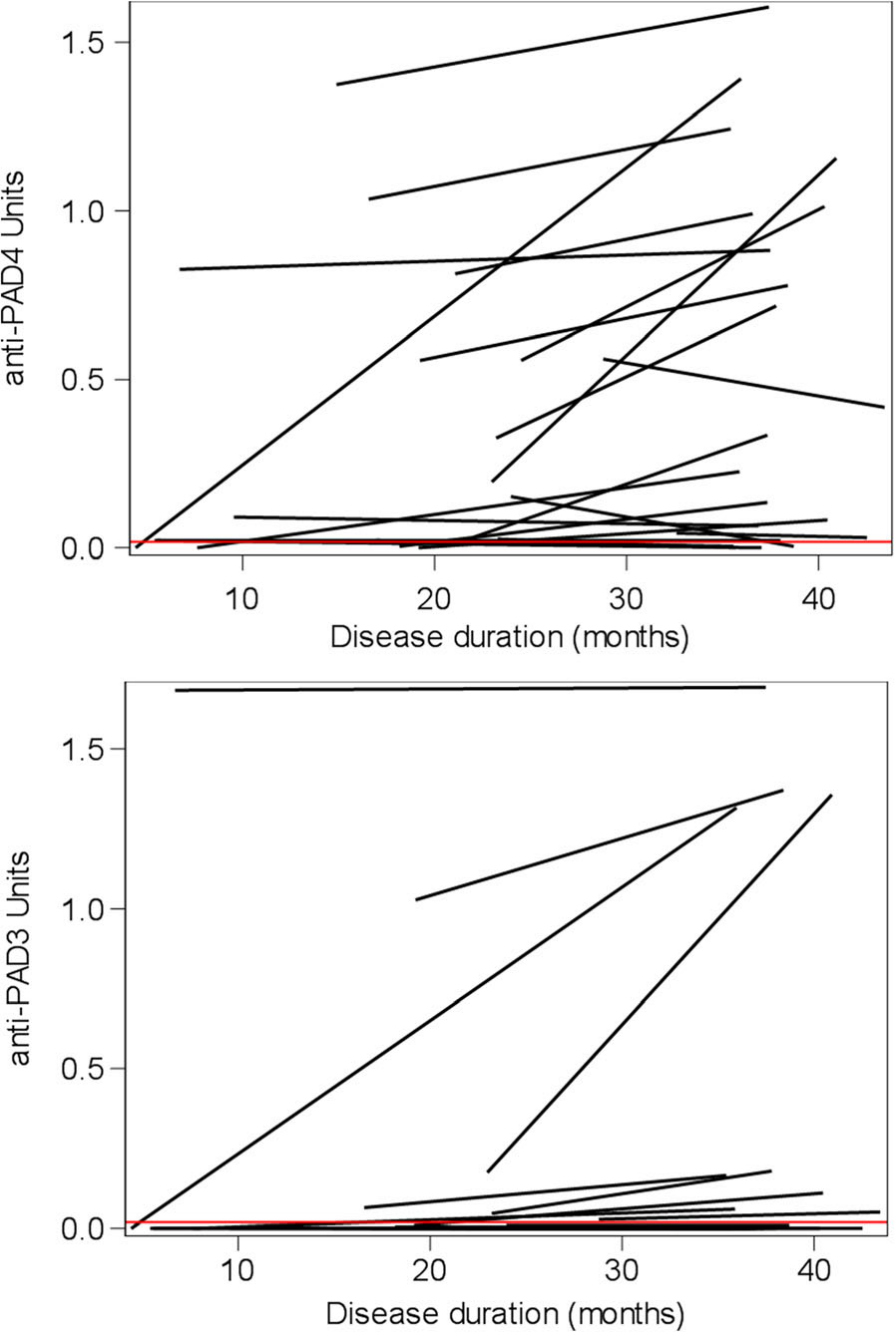
Changes in titers of anti-PAD4 antibodies and anti-PAD4/PAD3 cross-reactive antibodies among CLEAR I RA patients. Only patients who were anti-PAD4-positive (≥0.02 anti-PAD4 antibody IU) at any time (baseline or 36-month disease duration visit) were included (*N* = 21). Relative titers of anti-PAD4 antibodies (**a**) and of anti-PAD4 antibodies that cross-react with PAD3 (**b**) at baseline and 36 months of actual RA disease duration determined by densitometry. *Red horizontal line*, cutoff between low and high antibody titer. Changes in antibody levels from baseline to 36-month disease duration visit were tested using the Wilcoxon signed rank test, *P* < 0.05 considered significant. *PAD* peptidyl arginine deiminase enzyme (Color figure online)

Patients enrolled in CLEAR I, but not those enrolled in CLEAR II, had available longitudinal samples which allowed characterization of trends of anti-PAD4 and anti-PAD4/PAD3 antibodies over time. Only patients who were anti-PAD4-positive (≥0.02 anti-PAD4 antibody IU) at any time in the initial screen (*N* = 21) were included in this analysis. During the 36 months of disease, four patients seroconverted to anti-PAD4 positivity, with three of the four patients developing anti-PAD3 cross-reactive antibodies. Of the nine patients with low-titer antibodies against only PAD4 at baseline, four (44 %) became negative, three (33 %) remained low titer, and two (22 %) developed high-titer anti-PAD4 antibodies over 36 months. All eight patients who had high-titer anti-PAD4 antibodies at baseline remained high titer at 36 months of disease, with seven of eight demonstrating an increase in relative antibody titer. Of these eight patients, two had anti-PAD4 antibodies, whereas six had anti-PAD4/PAD3 cross-reactive antibodies for which the titer also increased over the 36-month period. In patients with early RA, both the overall anti-PAD4 and the anti-PAD4/PAD4 antibody titers significantly increased over time (*P* = 0.006 and *P* = 0.001, respectively) (Fig. 2a, b).

We performed separate analyses of radiographic severity in CLEAR I and CLEAR II, and found that the only significant variable was disease duration (data not shown). We then proceeded to combine the data of both cohorts to improve the power. Table 2 presents the results of the zero-inflated negative binomial models used to assess the relationship of these antibodies with higher radiographic scores. The association with higher radiographic scores detected in univariate analysis of anti-PAD4/PAD3 and ACPA positivity persisted after multivariable adjustment (Table 2). Results of the multivariable adjustment showed that anti-PAD4/PAD3 cross-reactive antibodies associated with higher radiographic scores with an IRR of 2.63 (95 % confidence interval (CI) 1.16, 5.95; *P* = 0.001), whereas anti-PAD4 antibodies alone did not associate with higher radiographic scores. Other variables included in the multivariable model that associated with higher radiographic score were disease duration and ACPA positivity. Radiographic severity reached statistical significance when former smokers were compared with the group who never smoked. This statistical significance was observed only in the anti-PAD4/PAD3 cross-reactive antibodies model (Table 2).

**Table 2:** Unadjusted and adjusted zero-inflated negative binomial models to evaluate the association of anti-PAD4 or anti-PAD4/PAD3 positivity with total radiographic scores for CLEAR I and CLEAR II patients at baseline (cross-sectional analysis) (*N* = 192)

Predictor/variable	Each variable combined with disease duration (BL) (IRR; 95 % CI)	Multivariable^a^ (BL) (IRR; 95 % CI)	*P* value (multivariable model)
Anti-CCP model			
Anti-CCP	**3.87 (1.90, 7.89)**	**4.48 (2.15, 9.36)**	**<0.001**
RA disease duration (per 6 months)	**1.05 (1.03, 1.07)**	**1.06 (1.04, 1.08)**	**<0.001**
Smoking (reference to never smoker)			
Current	1.36 (0.73, 2.50)	1.22 (0.67, 2.25)	0.517
Former	0.75 (0.39, 1.44)	0.57 (0.29, 1.20)	0.093
Anti-PAD4 model			
Anti-PAD4	1.25 (0.71, 2.22)	1.76 (0.89, 3.46)	0.102
RA disease duration (per 6 months)	**1.05 (1.03, 1.07)**	**1.05 (1.03, 1.07)**	**<0.001**
Smoking (reference to never smoker)			
Current	1.36 (0.73, 2.50)	1.40 (0.76, 2.58)	0.284
Former	0.75 (0.39, 1.44)	0.54 (0.25, 1.15)	0.110
Anti-PAD4/PAD3 model			
Anti-PAD4/PAD3 cross-reactivity	1.88 (0.88, 4.03)	**2.63 (1.16, 5.95)**	**0.020**
RA disease duration (per 6 months)	**1.05 (1.03, 1.07)**	**1.06 (1.04, 1.08)**	**<0.001**
Smoking (reference to never smoker)			
Current	1.36 (0.73, 2.50)	1.30 (0.71, 2.38)	0.403
Former	0.75 (0.39, 1.44)	**0.50 (0.25, 1.00)**	**0.049**

RA medication and age were not significantly associated with the outcome in the bivariable model and therefore were not included in the multivariable model

^a^Multivariable model adjusted for all the variables included in the table

*PAD* peptidyl arginine deiminase enzyme, *CLEAR* Consortium for the Longitudinal Evaluation of African Americans with Early Rheumatoid Arthritis, *BL* baseline data, *IRR* incidence rate ratio, *CI* confidence interval, *RA* rheumatoid arthritis

*P* values less than 0.05 are shown in bold

A total of 81 radiographic scores were used in the analysis of the longitudinal data. Additional file 1: Table S1 presents the characteristics of the CLEAR I cohort used for analysis of radiographic progression. Table 3 presents the results of radiographic progression between baseline and 36-month disease duration visit for the CLEAR I patients. Neither anti-PAD4 nor anti-PAD4/PAD3 positivity was associated with radiographic progression; the baseline radiographic score was the only factor associated with radiographic progression.

**Table 3:** Multivariable logistic regression models evaluating change in radiographic score (change of 0 or >0) between baseline (< 2 years disease duration) and approximately 36-month disease duration visit in CLEAR I patients (*N* = 81)

Predictor/variable	Odds ratio (adjusted)^a^	95 % CI	*P* value
Multivariable model 1: anti-CCP			
Anti-CCP	2.73	(0.83, 9.0)	0.099
RA disease duration (per 6 months)	0.90	(0.58, 1.38)	0.618
Baseline radiographic score (0 referent to >0)	**3.91**	**(1.38, 11.03)**	**0.010**
Smoking (reference to never smoker)			
Current	0.80	(0.25, 2.49)	0.695
Former	0.67	(0.18, 2.45)	0.546
Multivariable model 2: anti-PAD4			
Anti-PAD4	0.59	(0.14, 2.51)	0.609
RA disease duration (per 6 months)	0.87	(0.57, 1.33)	0.526
Baseline radiographic score (0 referent to >0)	**5.41**	**(1.97, 14.86)**	**0.001**
Smoking (reference to never smoker)			
Current	0.65	(0.21, 2.10)	0.478
Former	0.60	(0.17, 2.20)	0.445
Multivariable model 3: anti-PAD4/PAD3			
Anti-PAD4/PAD3	0.21	(0.02, 2.76)	0.235
RA disease duration (per 6 months)	0.87	(0.57, 1.35)	0.540
Baseline radiographic score (0 referent to >0)	**6.36**	**(2.22, 18.20)**	**<0.001**
Smoking (reference to never smoker)			
Current	0.55	(0.18, 1.78)	0.319
Former	0.51	(0.14, 1.89)	0.313

RA medication and age were not significantly associated with the outcome in the bivariable model and therefore were not included in the multivariable models

^a^Adjusted for all variables included in the table

*CLEAR* Consortium for the Longitudinal Evaluation of African Americans with Early Rheumatoid Arthritis, *CI* confidence interval, *RA* rheumatoid arthritis, *PAD* peptidyl arginine deiminase enzyme

*P* values less than 0.05 are shown in bold

## Discussion

Our study is the first assessment of anti-PAD4 and anti-PAD4/PAD3 cross-reactive antibodies in African-American patients with both early and established RA. The prevalence of anti-PAD4 antibodies in these African-American patients with RA was similar to that reported for patients of European ancestry with RA [4, 6, 8]. We found that cross-reactive anti-PAD4/PAD3 antibodies associated with higher radiographic scores, whereas anti-PAD4 antibodies without anti-PAD3 reactivity did not. Furthermore, anti-PAD4 and anti-PAD4/PAD3 antibody titers increased at the subsequent visit among patients with early RA. Neither anti-PAD4 nor anti-PAD4/PAD3 antibodies were significantly associated with radiographic progression. However, this may be due to the preponderance of normal radiographs at baseline, the small sample size (*N* = 81), the early stage of the disease (<2 years’ disease duration at enrollment), and/or the relatively short time between baseline and follow-up radiographs. The finding of the importance of disease duration in our model underscores the likelihood of this explanation. In addition, previous studies that analyzed the prevalence and association of anti-PAD4 and anti-PAD4/PAD3 antibodies with radiographic progression had longer disease duration (mean disease duration of 8–21 years) [6, 8].

PAD4 is one of the five subclasses of PAD enzymes that catalyze the conversion of arginine residues to citrulline residues. This modification has been implicated in changes to the structure and function of the targeted protein described to break tolerance and trigger autoimmunity [22, 23]. A subset of antibodies against PAD4 can increase calcium sensitivity that leads to activation of this enzyme and a subsequent increase in citrullination [6]. The origin of autoantibodies against PADs remains unknown, but high levels of PAD4 in the lungs and periodontium that occur in smokers implicate the potential importance of these sites [24]. Additionally, cross-reactive anti-PAD4/PAD3 antibodies have been associated with the development of interstitial lung disease among patients with RA [8, 23, 24], especially in patients with a history of smoking [8].

RA patients who smoke have been reported to have a higher risk for development of radiographic erosions [25]. We did not observe an association between smoking and radiographic severity and radiographic progression. However, the point estimates showed an increased risk for radiographic severity and a trend towards an association in the cross-sectional analysis. These differences were likely in part due to the small sample size in our study compared with larger epidemiologic studies.

Our study is the first to describe the pattern of anti-PAD4/PAD3 cross-reactive antibody titers over time, an aspect that has only thus far been described cross-sectionally [6, 8, 9]. One small study did analyze anti-PAD4 antibodies titers, but not the PAD3 cross-reactive subset, longitudinally in a predominantly Caucasian population with a mean RA disease duration of 11.5 years [13]. In that study, the anti-PAD4 antibodies were stable over 1 year of follow up. Unlike previously reports, the mean levels of anti-PAD4 antibodies and anti-PAD4/PAD3 antibody titers did increase significantly over a mean period of 22 months of follow up in our African-American cohort of patients with early RA (mean disease duration at enrollment of 15 months). In our cross-sectional model, anti-PAD4/PAD3 antibodies significantly associated with radiographic severity. This association persisted even after controlling for disease duration, which was the only variable associated with radiographic severity when the models for radiographic severity were run separately for each cohort. This suggests that a significant increase in radiographic severity may, albeit relatively small, occur when these antibodies target both PAD3 and PAD4; and that these antibodies are a risk factor for disease severity which accumulates over time. It has been reported that the development of anti-PAD4 antibodies occurs mainly in long-standing RA rather than in early RA. In our cohort, however, the majority of the patients positive for these antibodies had these antibodies even within the first 2 years of disease onset (CLEAR I data only).

It is possible that PAD4 (and therefore anti-PAD4 antibodies) has an effect on cytokine expression that influences disease activity. Indeed, PAD4 inhibition significantly attenuated the expression of neutrophil chemotactic proinflammatory cytokines (macrophage inflammatory protein-2 and keratinocyte-derived cytokine) in response to renal ischemia/reperfusion injury in a murine model [26]. This information is important because it suggests these antibodies may not only serve as a marker of disease severity but also have diagnostic value and could be a target of future therapies for RA.

This study has several strengths, including being the first analysis of anti-PAD4 and anti-PAD4/PAD3 cross-reactive antibodies in a well-characterized cohort of African- American patients with RA. This study also describes the longitudinal pattern of anti-PAD4 and anti-PAD4/PAD3 antibody titers in patients with early RA, the former of which has only been studied previously in patients with established RA [13]. A recent study of 39 patients with RA also found that anti-PAD4/PAD3 cross-reactive antibodies were strongly associated with joint erosions [27]. Limitations of this study include the relatively small sample size and that the longitudinal data were derived predominantly from patients with early RA with normal radiographs. We speculate that the lack of association between anti-PAD4 and anti-PAD4/PAD3 antibodies with radiographic progression in CLEAR I is mainly due to the small sample size and less likely due to a lack of contribution of these antibodies to more severe and aggressive RA. Notably, the effect of biologics on radiographic severity and progression was limited in this study because these medications had just been approved when the CLEAR Registry was established. This study could not assess accurately the radiographic effect of disease activity because the levels of several important variables used to measure disease activity, such as CRP, were not collected consistently, and disease activity instruments, such as the Disease Activity Score in 28 joints (DAS28), was not yet the most commonly used tool for disease activity.

## Conclusion

This is the largest and first study to analyze the relationship between radiographic severity and anti-PAD4 and anti-PAD4/PAD3 antibodies in African Americans. The prevalence of these antibodies was similar to that observed in cohorts of predominantly Caucasian or Native-American patients with RA. This analysis suggests that autoantibodies against PAD4 and PAD3 proteins may serve as biomarkers for identifying African-American patients with RA and higher radiographic severity. Future studies with larger cohorts are needed to accurately determine whether anti-PAD4 or anti-PAD4/PAD3 antibody titers associate with radiographic progression.

## Additional file

Additional file 1: Table S1. Characteristics of the CLEAR I patients used to analyze radiographic progression (*N* = 81). (DOCX 14 kb)

## Abbreviations

ACPA: Anti-citrullinated protein antibody
CLEAR: Consortium for the Longitudinal Evaluation of African Americans with Early Rheumatoid Arthritis
CRP: C-reactive protein
ELISA: Enzyme-linked immunosorbent assay
IRR: Incidence rate ratio
PAD: Peptidyl arginine deiminase enzyme
RA: Rheumatoid arthritis
