# Supplementary material for: Association of anti-peptidyl arginine deiminase antibodies with radiographic severity of rheumatoid arthritis in African Americans
Source: Arthritis Res Ther. 2016 Oct 22;18:241. doi: 10.1186/s13075-016-1126-7 (PMC5075170; doi:10.1186/s13075-016-1126-7)
Supplement: Additional file 1: Table S1. — Characteristics of the CLEAR I patients used to analyze radiographic progression (N = 81). (DOCX 14 kb) [file 13075_2016_1126_MOESM1_ESM.docx]

**Table S1** CLEAR I patient characteristics used to analyze radiographic progression (N = 81).

| Characteristic | Measure |
| --- | --- |
| Age, mean (SD) | 51.9 (11.6) |
| Disease duration (mo), mean (SD) | 15.8 (7.0) |
| Months between baseline and 36-month visit, mean (SD) | 22 (7.3) |
| Range of months between baseline and 36-month visit (min-max in months) | 10 - 42 |
| Total radiographic score at baseline, median (IQR) | 0 (0-1) |
| Proportion of total radiographic score (at baseline) |  |
| 0, (n) | 52 |
| >0, (n) | 29 |
| Total radiographic score at 36-month visit, median (IQR) | 1 (0-6) |
| Total radiographic score change from baseline to 36-month visit, median (IQR) | 0 (0-3) |
